# The Role of Virtual Reality on Parkinson’s Disease Management: A Bibliometric and Content Analysis

**DOI:** 10.3390/s25051432

**Published:** 2025-02-26

**Authors:** Qiang Wu, Mengli Qiu, Xiaomei Liu, WanJiaAaron He, Ting Yang, Chengsen Jia

**Affiliations:** 1 Rehabilitation Medicine Center and Institute of Rehabilitation Medicine, West China Hospital, Sichuan University, Chengdu 610041, China; wuqiang59@126.com (Q.W.); liuxiaomei@scu.edu.cn (X.L.); hxtfyt@163.com (T.Y.); 2 Key Laboratory of Rehabilitation Medicine in Sichuan Province, West China Hospital, Sichuan University, Chengdu 610041, China; 3 The First Clinical School of Guangzhou University of Chinese Medicine, Guangzhou 510000, China; mengli992021@163.com; 4 School of Public Health, LKS Faculty of Medicine, University of Hong Kong, Hong Kong 999077, China; aaronhe@hku.hk

**Keywords:** virtual reality, Parkinson’s disease, bibliometric, hotspots, research trends

## Abstract

The management of Parkinson’s disease (PD) has increasingly focused on innovative technologies, particularly virtual reality (VR), which has emerged as a significant tool for addressing neurological disorders. This bibliometric analysis summarizes current research trends and hotspots regarding VR applications in PD management. A comprehensive search of the Science Citation Index Expanded (SCIE) within the Web of Science Core Collection (WoSCC) identified 475 publications from 2000 to 2024. Key findings indicate a substantial increase in publication output, especially after 2013, driven by technological advancements and investments from major IT companies. Prominent research institutions and scholars from Australia, Israel, Italy, and Spain have led this field, exploring various VR applications for PD patients. The focus of VR therapy research has evolved from primarily addressing freezing of gait (FOG) to a broader range of functional impairments, including balance, postural control, upper limb motor, and cognitive function. This study provides valuable insights into the evolving landscape of clinical research on VR in PD management, highlighting global trends and potential areas for future investigation and application of VR therapies.

## 1. Introduction

Parkinson’s disease (PD) ranks as the second-most prevalent neurodegenerative disorder among the elderly, following Alzheimer’s disease [1]. Clinically, PD is characterized by motor dysfunctions, including resting tremors, bradykinesia, and postural instability [2]. Beyond these motor symptoms, patients frequently experience a variety of non-motor symptoms, including olfactory dysfunction, sleep disturbances, constipation, depression, anxiety, and cognitive impairments [3]. As the disease advances, both motor and non-motor functional deficits can severely diminish the patient’s quality of life [4]. While pharmacological treatment remains the primary intervention for PD, its effectiveness typically wanes after a few years, and many symptoms remain unresponsive to medication [5]. Consequently, exercise and cognitive training have emerged as vital adjunct therapies that positively impact symptom management [6]. However, traditional rehabilitation methods can be monotonous, posing challenges to sustained patient engagement.

Virtual reality (VR) technology, which simulates immersive environments and enables real-time interaction through visual, auditory, and tactile feedback, initially found its footing in the gaming industry [7]. Recent studies have explored VR’s potential as a rehabilitation tool, particularly for patients with neurodegenerative conditions. Over the past decade, VR technology has advanced rapidly, establishing itself as an innovative approach to rehabilitation [8]. Its key features—immersion, interactivity, and imaginative engagement—render it particularly suitable for PD rehabilitation. By creating captivating virtual environments and incorporating gamified elements, VR enhances patient motivation and adherence to high-frequency, intense functional training [9]. This innovative approach not only enhances the efficiency of rehabilitation processes but also provides a safe, convenient, and cost-effective long-term training solution for patients [10].

Research on the application of VR in PD rehabilitation has proliferated globally. For instance, a study by Feng et al. demonstrated that a 12-week VR training program resulted in significant improvements in balance, gait function, and mobility compared to conventional physical therapy [11]. Similarly, Cikajlo et al. found that VR training provided greater enjoyment and engagement for PD patients and led to more efficient functional improvements than traditional 2D exercise games [12]. Furthermore, Maggio et al. assessed the effects of VR on cognitive and behavioral recovery in PD patients, revealing that VR was notably more effective than traditional cognitive training in enhancing executive and visuospatial functions [13]. Although some studies have indicated that VR can improve balance and gait [14], not all research supports its superiority over conventional rehabilitation methods [15]. These findings suggest that VR represents a novel therapeutic avenue for PD rehabilitation, optimizing motor learning in a safe environment and aiding patients in improving balance, gait, cognitive function, and daily living activities [15]. Despite the growing body of literature on VR therapy in this context, much of it remains complex and unstructured, lacking clear applications. Furthermore, no bibliometric analysis has provided a comprehensive overview of VR’s role in managing PD. This study intends to clarify the developmental trajectory of VR in the field of PD; to identify the most active countries, research institutions, and authors, along with their respective research foci; and to characterize the evolution of research themes and future trends within this domain.

In response, this study aims to conduct a bibliometric analysis of global scientific outputs related to VR in PD management from its inception to 2024. Utilizing software such as VOSviewer (v.1.6.19) and CiteSpace (v.6.1.R1), we will systematically analyze publications from the Web of Science Core Collection (WoSCC) database. This analysis seeks to illuminate research hotspots and emerging trends in the application of VR for PD management, thereby guiding clinical practice and informing future research directions.

## 2. Methods

### 2.1. Data Sources and Search Strategy

**Figure 1:**
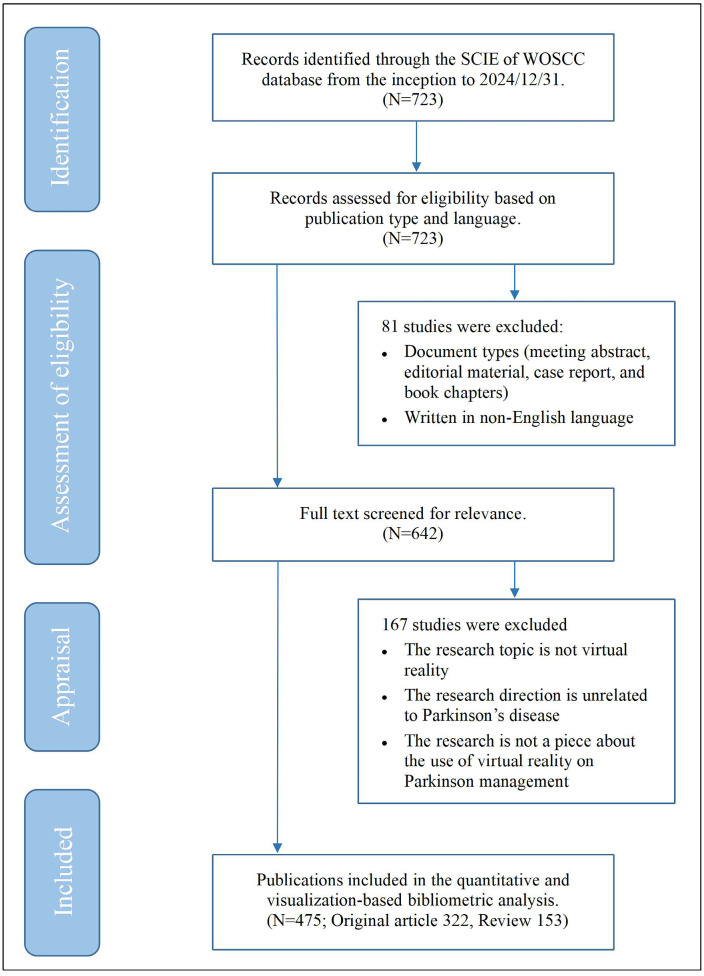
Flow chart of the bibliometric search and analysis process.

The literature data were sourced from the Science Citation Index Expanded (SCIE) within the WoSCC database. Web of Science (WoS), a globally recognized database of high-quality journals across numerous disciplines, particularly in the medical and health domain, is ideally suited for this research. A specific search strategy was employed using the following query: “(((parkinson)) OR (PD)) OR (parkinson’s disease)) AND ((Virtual reality) OR (VR))” on 31 December 2024. The retrieval period spanned from the inception of the database to the present date. The search was restricted to English-language publications and included reviews and original articles. Studies that did not focus on the application of VR in PD were excluded from consideration. The initial search yielded 723 papers. After removing duplicates and a rigorous screening process conducted by two independent researchers—who assessed the titles, abstracts, and full texts—475 relevant papers were ultimately included, as shown in Supplementary Materials. The literature selection process is depicted in Figure 1.

### 2.2. Data Extraction and Analysis

Bibliometrics constitutes a quantitative analytical framework that systematically examines publication patterns and citation networks to delineate disciplinary dynamics and intellectual progress [16]. This approach generates multidimensional outputs encompassing descriptive statistics, co-occurrence network (keywords, geographical distributions, and authorship patterns), and institutional collaboration matrices, enabling rigorous exploration of evolutionary trajectories and emergent research frontiers within specialized domains.

Our implementation integrates three analytical platforms: VOSviewer for network visualization, CiteSpace for temporal trend analysis, and Bibliometrix through its Biblioshiny interface for scientometric computations. In practice, we extracted general information from each included publication, encompassing the title, journal, publication year, citation count, authors, country of origin, institution, and keywords. These data were then imported into Microsoft Office Excel for further analysis and graphical representation. CiteSpace was used to perform cluster analysis and generate burst keyword graphs based on the extracted keywords, thereby reflecting current research trends and hotspots. CiteSpace parameters were set as follows: time slicing (2000–2024), years per slice (1 year), term source (all selected), node type (selected individually), selection criteria (top 50 objects), and pruning (pruning sliced networks). In the generated maps, the color of each node represents its cluster affiliation, the node size reflects citation frequency, and the links between nodes indicate the strength of collaboration. Corresponding descriptions will accompany all other figures and tables.

## 3. Results

### 3.1. Publication Outputs and Growth Trend

**Figure 2:**
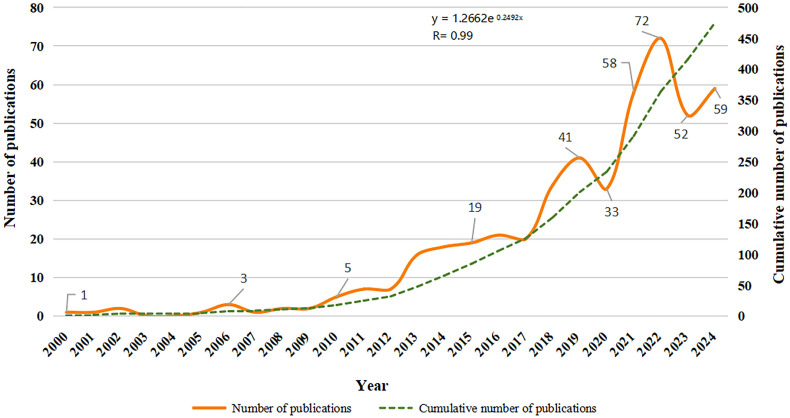
Trend of publication outputs on VR for PD management. The equation shown in the figure represents the results of the exponential regression for annual publication outputs over the past three decades.

This analysis encompasses 475 publications that meet the specified retrieval criteria. These publications include 322 original articles and 153 review articles, published between 2000 and 2024 (refer to Figure 2). The pioneering study in this field was conducted by D. Gourlay et al., who introduced a prototype networked virtual system aimed at assisting patients with neurological disorders, including PD, in cognitive and motor rehabilitation [17]. As illustrated in Figure 2 and Table 1, there is a discernible upward trend in annual research output, increasing from one publication in 2000 to 59 in 2024. Before 2012, the annual publication volume in this domain remained below ten articles. However, beginning in 2013, a significant escalation in publication volume occurred, likely attributable to the proliferation and decreasing costs of intelligent technologies, such as VR. This technological advancement has encouraged research institutions and hospitals to explore the integration of VR technology into the management of PD while maintaining cost-effectiveness. The year 2013 thus marks a pivotal turning point, indicating the gradual initiation of research focused on VR applications for PD patients. From 2021 to 2024, research in this area exhibited a slow growth trajectory, stabilizing after reaching its peak in 2022. This surge in research activity can be linked to the COVID-19 pandemic, which created conducive conditions and application scenarios for the remote rehabilitation and intelligent management of neurological disorders due to travel restrictions and social distancing measures. Consequently, research related to VR experienced significant growth during this period. Exponential regression analysis conducted in the present study revealed a strong correlation (R^2^ = 0.99) between annual publication outputs and the years over the past three decades. Furthermore, the mean total citations (TCs) per article × year were notably higher from 2013 to 2020, suggesting that the quality of articles published during this period was substantial, thereby supporting the hypothesis above.

### 3.2. National Distribution of Publications

**Figure 3:**
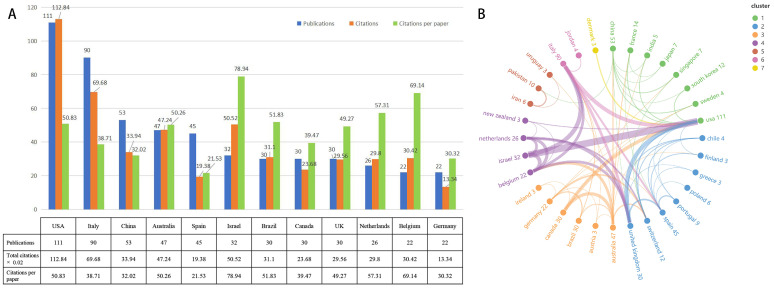
The top prolific countries and international collaboration network on VR for PD management research. (**A**) The number of publications, total citations, and citations per paper in the top 12 countries. (**B**) The cooperative network visualization map of countries.

The 475 articles within this field were distributed across 23 countries, with 12 countries contributing to 20 or more publications (see Figure 3A). The top five countries by publication volume are the United States (111 articles), Italy (90 articles), China (53 articles), Australia (47 articles), and Spain (45 articles). The United States leads in total citations (5642), followed by Italy (3484) and Israel (2526). Notably, Israel exhibits the highest average citations per article (78.94), followed by Belgium (69.14) and the Netherlands (57.31), suggesting that these countries have produced high-quality articles or significant foundational literature in this field. The national collaboration network (as depicted in Figure 3B) illustrates that the size of each node corresponds to publication volume, while the thickness of the connecting lines reflects the strength of collaboration between countries. The United States demonstrates the strongest collaboration with other nations, followed by the United Kingdom, Italy, and Israel. Specifically, the collaboration between the United States and Israel is particularly robust, indicating a deep partnership among researchers from these two countries. The international collaboration diagram also reveals a strong network centered around the United States, encompassing Asian countries such as China, Japan, and South Korea. This collaborative group primarily focuses on assessing and treating gross motor functions in PD patients (particularly gait, balance, muscle strength, and sensory integration) using VR technology, thereby reflecting the current research priorities in the field.

### 3.3. Institutional Contributions

**Figure 4:**
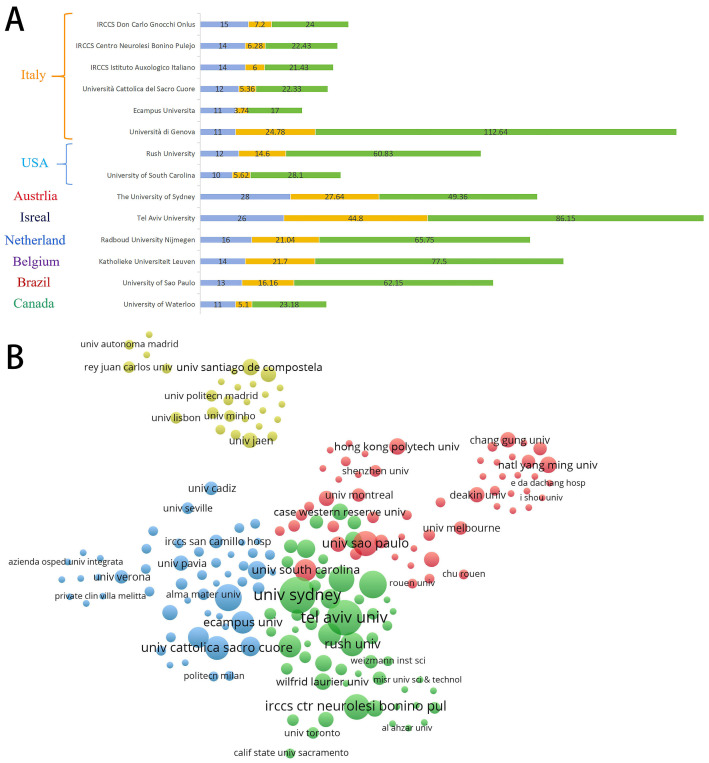
The top active institutions and the inter-institutional collaboration network on VR for PD management research. (**A**) The number of publications, total citations, and citations per paper in the top 14 institutions. (**B**) The cooperative network visualization map of institutions.

Recent advancements in VR technology have prompted significant interest in its application for PD research, with 869 institutions contributing to this emerging field. Notably, 14 institutions have published ten or more studies, as illustrated in Figure 4A. Among these, six institutions are located in Italy, two in the United States, and others are represented across various continents, including Europe (the Netherlands and Belgium), North America (Canada), Oceania (Australia), Asia (Israel), and South America (Brazil). This relatively uniform distribution of research indicates a global engagement with VR applications in PD, with the highest contributions stemming from institutions in Italy and the United States. The University of Sydney in Australia leads in publication volume with 28 articles, followed closely by Tel Aviv University in Israel with 26 articles and Radboud University Nijmegen in the Netherlands with 16 articles. In terms of total citations, Tel Aviv University ranks first with 2240 citations, followed by the University of Sydney with 1382 citations and the University of Genoa in Italy with 1239 citations. Furthermore, the University of Genoa and Tel Aviv University exhibit the highest average citations per article, at 112.64 and 86.15, respectively, highlighting their contributions to high-quality, peer-recognized research.

The institutional co-occurrence network depicted in Figure 4B reveals that Tel Aviv University demonstrates the strongest collaboration intensity within the field. Additionally, four major institutional clusters have emerged. The green cluster, primarily composed of Tel Aviv University and the University of Sydney, includes several European and American institutions that focus on assessing and treating gait—particularly the phenomenon of freezing of gait (FOG)—and balance in PD patients through VR interventions. The blue cluster consists of Italian institutions, including IRCCS research centers and various universities, which emphasize posture control and the application of VR for enhancing non-motor abilities in PD patients, such as cognitive functions (executive function, memory, and cognitive reserve), the quality of life, and psychological well-being. The red cluster comprises academic institutions from the United States and Asia, with a clinical focus. Researchers in this group employ various established immersive technologies, including Nintendo Wii, Kinect, and Oculus Rift, to PD patients for safety observations and specific efficacy comparisons. Additionally, a distinct cluster of academic institutions in Spain has emerged, where researchers are particularly dedicated to applying VR-based motor training techniques for upper extremity motor recovery in PD patients, with a specific emphasis on hand movements. Given their relatively recent engagement in this field, Spanish researchers have also conducted systematic reviews on the application of VR across diverse functional domains in PD, thereby providing more robust conclusions. This phenomenon indirectly reflects the current collaborative relationships among research institutions and highlights prevailing trends within this area of study.

### 3.4. Author Analysis

**Figure 5:**
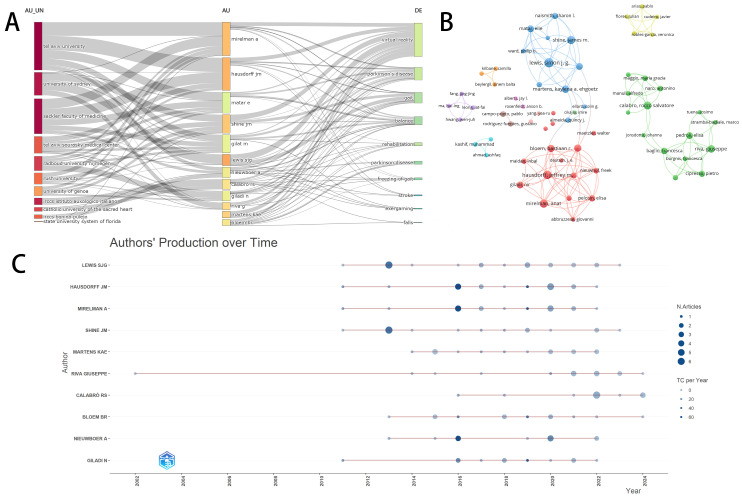
The top active authors and the collaboration network on VR for PD research. (**A**) The three-dimensional plots over the authors, their affiliations, and research directions. (**B**) Collaboration networks between authors. (**C**) Top 14 authors in terms of number of documents with their productivity over the time.

Regarding authorship, 2198 authors contributed to the 475 included studies. The data presented in Table 2 highlight 14 authors with more than ten publications, among which Simon J.G. Lewis and James M. Shine from the University of Sydney lead with 23 and 17 articles, respectively. Additionally, Jeffrey M. Hausdorff and Anat Mirelman from Tel Aviv University published 18 and 17 articles, respectively. Notably, the researchers from Tel Aviv University possess the highest citation counts, correlating with their collaborations on numerous high-quality studies. Figure 5A delineates the institutional affiliations of researchers and their primary research focus areas. An examination of the author collaboration network indicates that most authors have a limited number of publications and exhibit a relatively low level of collaborative engagement. However, a cluster of prolific authors emerges, comprising researchers from the University of Sydney, Tel Aviv University, and various IRCCS centers in Italy, as depicted in Figure 5B. These teams operate independently without significant inter-team collaboration. Figure 5C presents a temporal activity map of scholarly publications over the past three decades, where the circle diameter corresponds to the annual publication output. Researchers from the University of Sydney have primarily focused on FOG, with their research interest emerging around 2013, which catalyzed the rise of VR applications in PD research. In contrast, researchers from Tel Aviv University have emphasized enhancing overall motor capabilities, dual-task performance, and reducing fall risks in PD patients through VR technology since 2016. Unlike the groups above that focus on lower limb motor abilities, a notable number of recent publications have emerged from researchers at the Italian IRCCS centers, who investigate not only postural control but also the enhancement of non-motor abilities and the impact of these abilities on patients’ motor functions. These insights indicate the main directions within the field, with the latest research trends centering on the treatment of non-motor abilities in PD patients through VR.

### 3.5. Journal Characteristic

**Figure 6:**
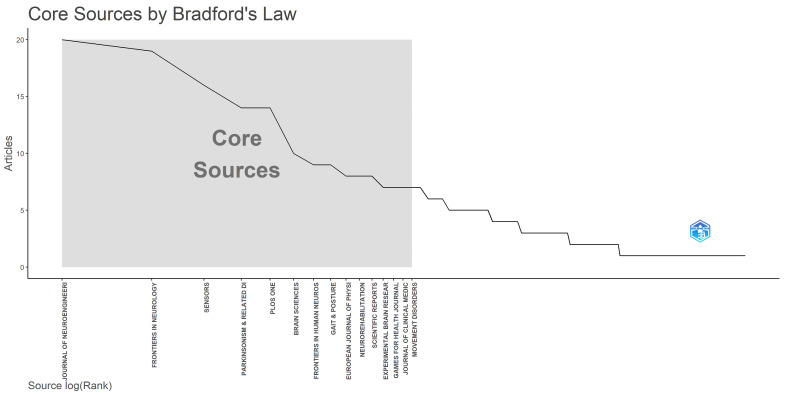
The related information of Bradford’s Law applied to the journals in this area.

The landscape of research in the domain of VR on PD is characterized by a diverse array of journals, with 198 journals publishing relevant studies. Notably, 11 of these journals have published more than eight articles, as detailed in Table 3. The leading journals by publication volume include the *Journal of NeuroEngineering and Rehabilitation* (20 articles, Impact Factor [IF] 5.2), *Frontiers in Neurology* (19 articles, IF 2.7), and *Sensors* (16 articles, IF 3.4). This distribution indicates a relatively fragmented journal ecosystem within this field. According to Bradford’s law, illustrated in Figure 6, 15 core journals have been identified, collectively publishing over one-third of all related papers, alongside 183 non-core journals.

Among these prominent publications, the *Journal of NeuroEngineering and Rehabilitation* stands out with the highest citation count, amassing 966 citations and an average of 48.30 citations per article. Conversely, while *The Lancet Neurology* has published only three articles, it boasts the highest overall citation count at 1230 citations, yielding an impressive average of 410.00 citations per article, reflective of its superior publication quality. However, it is noteworthy that the impact factors of leading journals in this domain are generally modest, with most ranging between 2 and 4. Only four journals are classified within the Q1 category of the Journal Citation Reports, indicating a need for improvement in the quality of significant journals in this field, which may, in turn, boost researchers’ enthusiasm for further exploration. Moreover, the majority of current journals fall under the categories of Multidisciplinary Sciences or Neurosciences, with limited publications in specialized areas such as Medical Informatics or Computer Science (e.g., IEEE and JMIR). This suggests a gap in dedicated VR journals within the field, underscoring the necessity for researchers to engage more actively with these specialized journals and submit their VR-related articles on PD management.

### 3.6. Analysis of Highly Cited Studies

A total of 475 articles included in this analysis have been cited 17,260 times, with the ten most-cited works accounting for 3527 citations, representing 20.43% of the overall citation volume, as presented in Table 4. The most frequently cited article is authored by Holden, published in 2005 in *Cyberpsychology & Behavior* (IF 4.2), which has garnered 728 citations [18]. This pivotal study reviews the application of VR in the motor rehabilitation of patients with neurological disorders, emphasizing the potential advantages of VR devices over conventional therapies. Notably, three highly cited studies [19,20,21] by Mirelman have significantly contributed to the field. In 2011, Mirelman et al. introduced a treadmill equipped with virtual obstacles, demonstrating that this innovative approach significantly improved gait speed and the ability to navigate ground obstacles in patients compared to traditional physical training [19]. In a subsequent study in 2016, a clinical trial conducted across five countries examined the safety of VR treadmill training for PD patients, revealing a significantly lower fall rate in the VR group relative to those using only a standard treadmill [20]. In 2019, Mirelman provided a comprehensive summary of assessment and treatment strategies for gait disturbances in PD patients, offering valuable insights into future research directions [21]. Collectively, these articles have played a crucial role in advancing the discipline.

### 3.7. Analysis of Keywords

**Figure 7:**
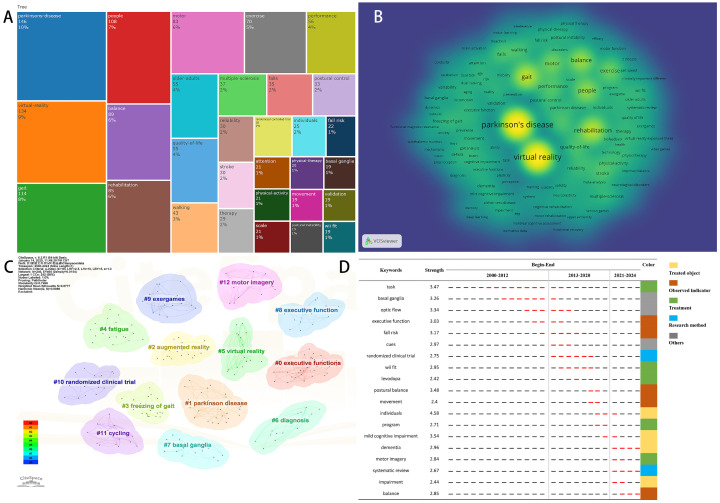
Analysis of keywords on VR for PD management field. (**A**) The main 30 keywords and frequency. (**B**) The keyword co-occurrence network map. (**C**) The keyword co-occurrence cluster map. (**D**) The top 19 keywords with the strongest citation bursts. The red line represents the period during which the keyword experienced a burst.

Keywords serve as a concise summary of a paper’s content, and high-frequency or emerging keywords can reflect current themes and anticipate future research trends. A keyword co-occurrence network comprising 217 nodes and 5827 links was created. Figure 7A represents the frequency of occurrence of different keywords in the included studies while Figure 7B displays the distribution of keyword prominence, with prominence increasing sequentially from blue to green to yellow. These figures revealed that the most commonly used keywords include “Parkinson’s disease” (frequency 146), “virtual reality” (frequency 134), “gait” (frequency 114), “people” (frequency 108), “balance” (frequency 89), “rehabilitation” (frequency 85), and “motor” (frequency 82). This distribution suggests that contemporary research predominantly concentrates on various immersive technologies, such as Wii Fit, Kinect, and augmented reality, applied to training methodologies (including exercise, motor imagery, and rehabilitation) for PD patients facing cognitive impairments (such as mild cognitive impairment and dementia) or motor dysfunctions (including FOG and falls). Randomized controlled trials (RCTs) and systematic reviews are frequently employed to evaluate cognitive functions, executive functions, postural control, gait, and the quality of life in these populations. Notably, studies utilizing VR technology to enhance gait and balance in PD patients represent a significant trend in the current research landscape.

Using the logarithmic likelihood ratio (LLR) algorithm, thirteen clusters were identified with a Q-value of 0.7496 and silhouette values exceeding 0.5 for each cluster, thereby affirming the reliability and reasonableness of the clustering results (Figure 7C). The identified clusters can be categorized into several thematic areas: clusters #2, #5, and #9 encompass various immersive technologies employed in the management of neurological diseases, including serious games, video games, exergames, and augmented reality. Clusters #0, #8, #3, and #4 specifically highlight the primary applications of VR in managing PD populations, particularly focusing on executive function, lower limb motor function (balance and gait), and upper limb motor function. Cluster #6 underscores the significance of deep learning and machine learning techniques in differentiating populations with cognitive impairments. Clusters #11 and #12 delineate specific VR training modalities, such as cycling, motor imagery, and action observation, reflecting diverse avenues of research in the application of VR for PD rehabilitation.

Figure 7D illustrates the top 19 keywords exhibiting the strongest citation bursts. Among these, “task” emerged as the leading keyword with the longest duration (2005–2014), while “individuals,” “mild cognitive impairment,” and “postural balance” demonstrated the highest burst strength. Recent keywords such as “dementia,” “motor imagery,” “systematic review,” and “balance” indicate evolving trends and potential future research directions. In summary, the keyword analyses underscore the growing significance of VR technology in rehabilitating PD patients, revealing both established research themes and emerging trends that may shape future investigations in this vital area of study.

## 4. Discussion

### 4.1. Overview of the Results

This study presents a comprehensive investigation into the application of VR in the management of PD. Utilizing advanced software tools such as VOSviewer and CiteSpace, we visually represent the research developments within this domain. The trajectory of research can be distinctly categorized into two phases based on publication timelines. Prior to 2012, the application of VR technology in healthcare remained largely exploratory. During this period, researchers primarily concentrated on assessing the effectiveness of VR-based virtual scene gait training for patients with PD. Their findings supported the preliminary efficacy of VR and laid the groundwork for subsequent studies expanding VR applications to posture control and cognitive functions [19,22]. The continuous advancement of VR technology, coupled with the reduction in healthcare costs, has led to an increasing number of researchers directing their attention toward this area. Data in Figure 2 and Table 1 indicate a gradual rise in VR-related research following 2013, accompanied by significant citations from subsequent scholars. This trend suggests that 2013 may represent a pivotal year, marking the onset of explosive growth in research within this field. The rapid development and widespread adoption of smart technologies have likely contributed to the surge in VR applications. Additionally, the entry of major information technology companies into the VR sector presents substantial investment and growth opportunities [23]. Notable examples include Facebook Technologies’ acquisition of Oculus Rift in 2014, Microsoft’s launch of HoloLens and Windows Mixed Reality, and Sony’s release of the PlayStation VR device in 2016. As we approach the era of 5G technology, the potential for VR applications to further proliferate—particularly in healthcare and the management of neurological disorders—appears increasingly promising. The emergence of more portable and personalized VR devices seems imminent, indicating that future researchers should not only focus on medical aspects but also broaden their perspectives to encompass information technology and interdisciplinary development. This approach could catalyze a significant revolution in the healthcare sector.

The results of this study indicate that high-income countries such as North America, Europe, Australia, and Israel dominate the active research landscape, underscoring the correlation between scientific research development and economic conditions, as well as the academic atmosphere of research institutions. An intriguing observation is the clustering of prominent authors within specific research institutions and countries. The data suggest that the initial research surge in this field originated from scholars at the University of Sydney in Australia, drawing attention to the application of VR in addressing FOG. Following this, researchers from Tel Aviv University in Israel began to focus more on the effectiveness of VR in enhancing overall motor functions in PD patients. Building on these foundational studies, researchers from other countries and institutions have gradually expanded their inquiries to include other areas, such as VR interventions targeting upper limb motor functions (e.g., studies from Spain) and cognitive functions (e.g., research from Italy’s IRCCS institutions). However, there remains a notable lack of sufficient international or inter-institutional collaboration in this field. This phenomenon may stem from the field’s nascent developmental stage, where critical parameters including therapeutic efficacy, optimal application protocols, and mechanisms remain insufficiently characterized. Furthermore, the current industrial landscape demonstrates pronounced geographical concentration, with the majority of VR hardware manufacturers headquartered in western Europe and North America according to the VR Report 2024 [24], creating inherent imbalances in resource allocation and collaborative infrastructure. A prior scientometric analysis [25] revealed that international partnerships—except for robust U.S.–China collaborations—remain underdeveloped, a pattern consistent with our findings. As an increasing number of research institutions and personnel engage in VR-based research for PD, future studies are poised to feature multicenter, large-sample, high-quality designs, thereby providing valuable insights for clinical practice.

In terms of publication venues, the top ten prolific journals account for 28.42% of the total publications in this research domain. This indicates a broad distribution of papers across various journals, with a core group of journals yet to be established. Furthermore, the impact factors (IFs) of these active journals are relatively low, with only one journal achieving an IF above 5, suggesting a need for improved quality in these publications. Notably, the majority of articles in this field began to appear in general or neurology journals around 2010. In contrast, articles published in medical informatics or computer science journals (such as IEEE and JMIR) have mostly emerged after 2015, albeit in limited numbers, highlighting a deficiency in specialized VR journals. As interdisciplinary research deepens and expands, it is anticipated that an increasing number of medical informatics journals will emerge, providing researchers in this field with more platforms to showcase high-quality findings.

### 4.2. Keyword and Trend Analysis

Keyword analysis offers a visual representation of the prevailing themes and their interrelationships within a specific domain. Based on the clustering and burst word graphs, four prominent areas of VR application for PD patients can be identified: FOG, balance and posture control, cognitive function, and upper limb motor function. This underscores the primary applications of VR technology in managing PD.

Freezing of Gait: FOG is a prominent manifestation of motor dysfunction in patients with PD, characterized by asymmetry and a periodic absence or reduction of forward movement despite the patient’s intention to walk [26]. Recent advancements in VR technology offer innovative approaches for assessing FOG in PD patients by simulating real-life environments that trigger freezing episodes. Matar et al. found that VR could effectively differentiate between patients with and without FOG, revealing that those with FOG exhibited significantly longer step delays in the “off” state compared to the “on” state [27]. Current treatment modalities for FOG primarily include pharmacological interventions and deep brain stimulation. However, static VR technology has been shown to enhance walking speed and stride length, thereby improving overall walking efficiency in PD patients [28]. Improvements in gait are primarily driven by feedforward learning mechanisms, enabling VR to augment patients’ responses to external perturbations or modified environmental conditions. This augmentation strengthens feedforward motor control, particularly during dual-tasking or low-sensory feedback situations.

PD patients often experience deficits in higher central nervous system functions, leading to diminished perceptual processing capabilities. VR rehabilitation techniques can integrate visual and auditory stimuli, immersing patients in therapeutic environments that aim to alleviate FOG [29,30].

Balance and Posture Control: Balance disorders are frequently compromised in PD patients, primarily due to abnormal postural control, resulting in bradykinesia that severely impacts their quality of life. VR training can facilitate the development of feedforward strategies and provide adequate sensory feedback, maximizing motor balance capabilities [29,31]. Stozek et al. demonstrated that systematic rehabilitation training significantly improved balance and body rotation abilities in PD patients [32]. Building on this, VR technology can accelerate the center of mass oscillation speed, substantially enhancing movement amplitude [33]. By incorporating engaging gaming tasks, VR can adjust the coordination of the trunk and limbs, improving the control of the ankle, knee, and hip joints, ultimately maximizing balance retention [34]. Research indicates that virtual gaming operations are effective for addressing coordination impairments in PD patients [35]. According to motor learning theories, repeated and sustained stimuli from virtual environments serve as a beneficial approach to counteract motor behavior degradation and balance dysfunction in these patients [36,37]. The efficacy of VR technology in improving balance among PD patients is widely recognized, with current studies focusing on determining optimal treatment dosages.

Cognitive Function: Cognitive impairments in PD patients manifest primarily as deficits in memory, attention, and executive function, significantly influencing their motor behaviors. Moreover, through various game designs, VR rehabilitation can facilitate brain function compensation and reorganization. This process enhances patients’ information processing capabilities and supports the formation of normal functional patterns, ultimately promoting recovery from cognitive impairments [38]. Hajebrahimi et al. [39] found that VR-based motor games significantly improved visual memory delay, overall cognition, and performance on the Boston Naming Test in PD patients, with fMRI indicating increased activity in the precuneus post-training. Cheng et al. combined repetitive transcranial magnetic stimulation (rTMS) with VR training, observing notable improvements in delayed memory scores and MoCA visuospatial/executive function scores compared to rTMS alone [40]. A systematic review by Triegaardt et al. highlighted that VR interventions significantly enhanced cognitive function in PD patients [41]. However, due to the limited literature available, further research is needed to comprehensively evaluate the effectiveness of VR on cognitive function in PD patients.

Upper Limb Motor Function: Upper limb motor function in PD patients often presents with resting tremors, agility impairments, and deficits in muscle strength and flexibility, complicating daily activities [42]. While pharmacological treatments can alleviate some primary symptoms, the response of hand function impairments to medication may be limited. Fernandez applied a novel immersive VR technology designed for serious gaming to 23 PD patients, revealing significant improvements in muscle strength, finesse, overall coordination, and movement speed on the affected upper limb, coupled with high patient satisfaction and adherence [43]. This finding is corroborated by other studies, including Cikajlo et al., which indicated that PD patients receiving treatment with 3D Oculus Rift exhibited better upper limb motor function and reduced tremor symptoms compared to those treated with liquid crystal display technology [12]. These improvements may be attributed to VR’s role in enhancing proprioceptive integration, thereby facilitating more effective planning and execution of voluntary movements and improving upper limb motor function [44]. However, the literature on this research direction has been limited and primarily concentrated in recent years, necessitating further investigation to establish definitive conclusions. A summary of Section 4 can be found in Table 5.

### 4.3. Mechanisms Underlying VR-Based Rehabilitation

The mechanisms underlying VR-mediated rehabilitation are increasingly understood. From a motor learning perspective, VR-based training demonstrates high-intensity, task-oriented, and multisensory feedback characteristics that simultaneously stimulate patients’ visual, auditory, and somatosensory systems. Through immersive or semi-immersive virtual environments, this modality enhances patient engagement in rehabilitation processes, thereby significantly improving treatment adherence. By simulating functional training scenarios and activities of daily living, VR facilitates neuromuscular memory consolidation and movement coordination, ultimately enhancing postural control and global motor function [12,40]. Beyond motor domains, VR enables environment enrichment and diversified task design that activates higher-order cognitive processing and strategic planning, thereby modulating cortical reorganization patterns in cognitive-emotional regulation circuits [22,45]. Collectively, the synergistic integration of customizable task complexity, multisensory stimulation across visual, auditory, and multiple brain regions establishes VR as a paradigm-shifting modality that transcends traditional rehabilitation constraints.

### 4.4. Summary of VR Intervention on PD

In the field of rehabilitation, particularly concerning the motor and cognitive rehabilitation of PD patients, the application of VR technology for assessment and treatment represents an innovative and effective approach. Patients can select the type of VR that best suits their needs and goals, whether immersive, semi-immersive, or non-immersive. Utilizing high-quality VR equipment can significantly enhance the efficacy of assessments and treatments and the overall user experience. Task design for cognitive assessment and training should encompass various cognitive domains, including memory, spatial cognition, and executive function. VR interventions targeting motor function should aim to simulate real-life scenarios, such as shopping, household management, and daily activities. The integration of VR with multiple emerging technologies (e.g., artificial intelligence, telemedicine, and wearable sensors) represents a discernible trend [46,47]. The COVID-19 pandemic has further accelerated the adoption of VR in telemedicine [48]. Future PD patients may receive personalized assessments and treatments within community settings while providing real-time feedback to specialists for tailoring individualized rehabilitation plans. Such approaches could enhance clinical efficiency and ensure patients receive appropriate therapies under monitoring, potentially defining future healthcare paradigms [49].

Despite the substantial potential of VR technology in exploring mechanisms and rehabilitation for PD patients, challenges remain. Numerous VR training devices and games, such as Nintendo Wii, Xbox Kinect, Wordplay VR, and other customized VR rehabilitation systems for PD patients, exist. However, current research has not identified which type of training device, game, or training regimen is most suitable. A study by Alves et al. compared the efficacy of Nintendo Wii and Xbox Kinect for PD patients, revealing that only Nintendo Wii demonstrated improvements in both single-task and dual-task gait functions [50]. Adverse events to VR during rehabilitation training are rarely reported. Albani et al. noted instances of visual hallucinations induced by VR, which were associated with prolonged reaction times and decreased stimulus discrimination abilities, as well as an increased risk of dementia in affected patients [51]. Previous VR-related trials [52,53] have documented adverse reactions in patients, including cybersickness, disorientation, and eye strain, which warrant serious consideration. Currently, VR therapy remains relatively high cost and not yet suitable for widespread adoption. However, with the continuous advancement of VR technology, the increasing availability of cost-effective hardware devices, and the reusable nature of patient-specific VR software, VR therapy is anticipated to experience rapid development [54,55]. Beyond these factors, the successful clinical implementation of VR therapy still faces multiple barriers, including various technical limitations in both software and hardware that require resolution to meet clinical demands and personalized requirements. Additional considerations encompass the treatment-response specificity across diverse populations, as well as the acceptance and feasibility of VR technology among elderly patients. Furthermore, the confidentiality of personal data, as well as ethical and political considerations, must be systematically incorporated into subsequent experimental designs. Future research should first establish the safety profile of VR applications in PD management. Investigations could prioritize enhancing gameplay engagement and developing personalized gaming configurations. Rigorous large-scale, multicenter, double-blind clinical trials with appropriate control groups are essential to minimize bias. Furthermore, comprehensive evaluation metrics—including psychological function and the quality of life—should be explored to demonstrate VR therapy’s effectiveness across multiple domains in PD patients.

### 4.5. Strength and Limitations

This study represents the first bibliometric analysis summarizing the current publications and trends regarding VR in the management of PD over the past 30 years. We employed widely recognized bibliometric tools such as CiteSpace and VOSviewer to quantitatively analyze specific data pertaining to countries, institutions, authors, journals, citations, and keywords, interpreting the results from multiple perspectives to provide valuable insights for researchers.

Nonetheless, this research has several limitations. First, while the WoS is regarded as a suitable academic database containing a wide array of publications relevant to our topic, other databases such as Medline, Embase, Scopus, and IEEE Xplore should also be considered for future research to improve the comprehensive of findings. Second, our review included only English-language papers classified as reviews or articles, potentially overlooking significant contributions in other languages. Third, the exclusive reliance on IF and Citation as evaluation metrics for papers and journals carries inherent biases, exemplified by disciplinary variability in citation practices, manipulable self-citation rates, the inadequate representation of emerging journals, and inherent time-lag effects with constrained temporal relevance. To address these challenges, future research should integrate multidimensional evaluation parameters such as H-index, Immediacy Index, and social media engagement metrics when appraising the academic value of the journal or research. Finally, bibliometrics does not address questions of efficacy but instead focuses on the overall framework of a field. It does not involve a professional quality assessment or clinical significance judgment of the included articles. Future investigations should clarify specific therapeutic mechanisms and parameters of VR therapy for PD patients through well-designed RCTs or systematic reviews, thereby aiding the development of clinical guidelines.

## 5. Conclusions

Our research primarily analyzes current research hotspots and trends in the application of VR technology for patients suffering from PD through various analytical techniques and software, guiding clinical practice and future research directions. Despite a consistent increase in publications related to VR treatment for PD over the past 30 years, a notable surge in published papers has been observed since around 2013, coinciding with substantial investments from IT companies and ongoing innovations in VR technology. Leading research institutions and authors from Australia, Israel, Italy, and Spain have dominated this field, focusing on diverse themes related to VR applications in PD. The scope of popular research topics has expanded from an initial emphasis on FOG to encompass a wider array of applications, including balance and posture control, cognition, and upper limb motor function. However, inter-country and inter-institutional collaboration in this field remains relatively sparse, and there is a lack of multicenter, large-sample, high-quality studies. Additionally, the prevailing journals in this research area exhibit moderate quality, with a scarcity of high-caliber publications in specific medical informatics. These findings may assist future researchers in better understanding the hotspots and evolving trends associated with VR in managing PD patients.

## Figures and Tables

**Table 1:** The number of articles and citations over the timespan 2000–2024.

Year	N	Mean TC per Art	Mean TC per Year	Year	N	Mean TC per Art	Mean TC per Year
2000	1	8.00	0.31	2013	16	94.06	7.24
2001	1	76.00	3.04	2014	18	72.28	6.02
2002	2	30.00	1.25	2015	19	62.05	5.64
2003	0	0.00	0.00	2016	21	89.14	8.91
2004	0	0.00	0.00	2017	20	60.95	6.77
2005	1	728.00	34.67	2018	34	45.29	5.66
2006	3	59.67	2.98	2019	41	54.73	7.82
2007	1	44.00	2.32	2020	33	39.73	6.62
2008	2	115.50	6.42	2021	58	17.50	3.50
2009	2	35.00	2.06	2022	72	12.39	3.10
2010	5	41.60	2.60	2023	52	6.98	2.33
2011	7	80.00	5.33	2024	59	1.76	0.88
2012	7	66.29	4.74				

N: number; TC: total citations; Art: article.

**Table 2:** The top 14 active authors published literature on VR for PD management.

Rank	Author	Institution	Country	N	TC	Citations per Art	H Index
1	Lewis, Simon J.G.	The University of Sydney	Australia	23	1074	46.70	74
2	Hausdorff, Jeffrey M.	Tel Aviv University	Israel	18	1821	101.17	107
3	Mirelman, Anat	Tel Aviv University	Israel	17	1798	105.76	43
4	Shine, James M.	The University of Sydney	Australia	17	966	56.82	24
5	Calabro, Rocco Salvatore	IRCCS Ctr Neurolesi Bonino Pulejo	Italy	15	318	21.20	49
6	Martens, Kaylena A. Ehgoetz	University of Waterloo	Canada	14	467	33.36	24
7	Riva, Giuseppe	IRCCS Istituto Auxologico Italiano	Italy	14	327	23.36	70
8	Nieuwboer, Alice	Catholic University of Leuven	Belgium	12	1087	90.58	68
9	Bastiaan Bloem	Radboud University Nijmegen	Netherlands	12	928	77.33	103
10	Giladi, Nir	Tel Aviv University	Israel	11	1241	112.82	101
11	Matar, Elie	The University of Sydney	Australia	11	768	69.82	19
12	Gilat, Moran	The University of Sydney	Australia	11	654	59.45	28
13	Pedroli, Elisa	IRCCS Istituto Auxologico Italiano	Italy	11	254	23.09	24
14	Baglio, Francesca	IRCCS Don Carlo Gnocchi Onlus	Italy	10	161	16.10	29

N: number; TC: total citations; Art: article.

**Table 3:** The top 11 most productive journals in the VR for PD management field.

Rank	Journal	N	IF	JCR	OA	TC	Citations per Art
1	*Journal of NeuroEngineering and Rehabilitation*	20	5.2	Q1	Yes	966	20
2	*Frontiers in Neurology*	19	2.7	Q2	Yes	512	19
3	*Sensors*	16	3.4	Q2	No	309	16
4	*Parkinsonism & Related Disorders*	14	3.1	Q2	No	638	14
5	*PLoS One*	14	2.9	Q1	Yes	458	14
6	*Brain Sciences*	10	2.7	Q3	Yes	77	10
7	*Frontiers in Human Neuroscience*	9	2.4	Q2	Yes	311	9
8	*Gait & Posture*	9	2.2	Q2	No	193	9
9	*Neurorehabilitation*	8	1.7	Q2	No	233	8
10	*European Journal of Physical and Rehabilitation Medicine*	8	3.3	Q1	No	229	8
11	*Scientific Reports*	8	3.8	Q1	Yes	66	8

N: number; IF: impact factor; JCR: journal citation reports. OA: open access journal; TC: total citations; Art: article.

**Table 4:** The top 10 most-cited papers in the VR for PD management field.

Rank	Title	Author (Year)	Journal (IF)	Type	TC
1	Virtual environments for motor rehabilitation: Review	Holden (2005)	*Cyberpsychology & Behavior* (4.2)	Review	728
2	Exercise-enhanced neuroplasticity targeting motor and cognitive circuitry in Parkinson’s disease	Petzinger (2013)	*Lancet Neurology* (46.6)	Review	514
3	Gait impairments in Parkinson’s disease	Mirelman (2019)	*Lancet Neurology* (46.6)	Review	404
4	Effectiveness of home-based and remotely supervised aerobic exercise in Parkinson’s disease: a double-blind, randomised controlled trial	Van der Kolk (2019)	*Lancet Neurology* (46.6)	Article	312
5	Addition of a non-immersive virtual reality component to treadmill training to reduce fall risk in older adults (V-TIME): a randomised controlled trial	Mirelman (2016)	*Lancet* (98.4)	Article	300
6	A meta-analysis and systematic literature review of virtual reality rehabilitation programs	Howard (2017)	*Computers in Human Behavior* (9.0)	Review	277
7	Virtual reality for gait training: can it induce motor learning to enhance complex walking and reduce fall risk in patients with Parkinson’s disease?	Mirelman (2011)	*Journal of Gerontology Series A Biological Sciences and Medical Sciences* (4.3)	Article	265
8	Rehabilitation for Parkinson’s disease: Current outlook and future challenges	Abbruzzese (2016)	*Parkinsonism & Related Disorders* (3.1)	Article	263
9	The role of exergaming in Parkinson’s disease rehabilitation: a systematic review of the evidence	Barry (2014)	*Journal of NeuroEngineering and Rehabilitation* (5.2)	Review	234
10	Effect of Nintendo Wii™-based motor and cognitive training on activities of daily living in patients with Parkinson’s disease: a randomised clinical trial	Pompeu (2012)	*Physiotherapy* (3.1)	Article	230

IF: impact factor; Art: article; TC: total citations.

**Table 5:** Synoptic summary of Section 4.

Category Description	Discussion of Results
Basic Information Analysis	
Phases of Research	Pre-2012: Exploratory phase focused on VR-based gait training for PD patients, validating preliminary efficacy. Post-2013: Explosive growth driven by VR advancements (e.g., Oculus Rift and HoloLens) and reduced healthcare costs. Key milestones: Corporate investments (Facebook, Microsoft, and Sony); 5G technology enabling broader healthcare applications.
Geographical and Institutional Insights	Dominant Regions: High-income countries (North America, Europe, Australia, and Israel) lead due to economic strength and academic infrastructure. Key Institutions: University of Sydney (Australia): Pioneered VR for FOG; Tel Aviv University (Israel): Focused on VR for motor function enhancement; Spain/Italy: Expanded to upper limb mobility and cognitive rehabilitation; Collaboration Gaps: Limited international partnerships; 78% of VR hardware manufacturers based in western Europe/North America.
Publication Analysis	Journal Distribution: Top 10 journals account for 28.42% of publications; low IF (only 1 journal >IF 5); From 2010 onward, the majority of articles have been published in general or neuroscience journals. Since 2015, publications have increasingly appeared in computer science/medical informatics journals (e.g., IEEE and JMIR). Need: Specialized VR journals and higher-quality interdisciplinary platforms.
Key Research Themes	
FOG	VR simulates real-world scenarios to assess FOG. Enhances gait and FOG via feedforward learning and sensory integration (visual/auditory stimuli).
Balance and Posture Control	VR games improve trunk–limb coordination and joint control (ankle, knee, and hip). Accelerates center-of-mass oscillation and movement amplitude. Repeated virtual stimuli counteract motor degradation (motor learning theory).
Cognitive Function	VR games enhance memory, attention, and executive function (e.g., Boston Naming Test and MoCA scores). VR boosts precuneus activity (fMRI evidence) and cognitive recovery.
Upper Limb Motor Function	Immersive VR (e.g., Oculus Rift) improves muscle strength, coordination, and tremor reduction through enhancing proprioceptive integration for voluntary movement planning.

VR: virtual reality; PD: Parkinson’s disease; FOG: freezing of gait; IF: impact factor.

## Data Availability

The raw data supporting the conclusions of this article will be found in Supplementary Materials.

## Supplementary Materials

The following supporting information can be downloaded at: https://www.mdpi.com/article/10.3390/s25051432/s1.

## Abbreviations

The following abbreviations are used in this manuscript:

VR	Virtual Reality
PD	Parkinson’s Disease
WoSCC	Web of Science Core Collection
SCIE	Science Citation Index Expanded
WoS	Web of Science
TC	Total Citations
FOG	Freezing of Gait
JCR	Journal Citation Reports
OA	Open Access Journal
RCT	Randomized Controlled Trial
LLR	Logarithmic Likelihood Ratio
IF	Impact Factor
rTMS	Repetitive Transcranial Magnetic Stimulation
